# Community Pharmacists’ Perceptions, Barriers, and Willingness for Offering Sexual and Reproductive Health Services

**DOI:** 10.3390/ijerph182010735

**Published:** 2021-10-13

**Authors:** Ali Mofleh Alshahrani, Mona Y. Alsheikh

**Affiliations:** Department of Clinical Pharmacy, College of Pharmacy, Taif University, Taif 26571, Saudi Arabia; a.mona@tu.edu.sa

**Keywords:** counselling, community services, sexual health, reproductive health, pharmacists, perception

## Abstract

The role of community pharmacists is crucial for promoting health and providing consultation related to sexual and reproductive health. This study measured the perception of community pharmacists in the Kingdom of Saudi Arabia (KSA) towards the provision of counselling services on sex education and reproductive health, including barriers to and proficiency in the delivery of services. A cross-sectional survey was developed and distributed electronically to pharmacists, and responses were analyzed using SPSS version 26. Graphical representations for various opinions on perception, proficiency and barriers were created. More than 80% of pharmacists placed a high value on counselling patients on sex and reproductive health, about 90% counselled their patients very often (74.6%) or often (22.2%), and 3.2% of pharmacists did not counsel patients. Most respondents believed counselling was very important (65.3%) or important (15.1%), with only 19.6% of respondents indicating it was not important. Barriers to offering services included fear from responsibility and liability (*M* = 4.8), lack of information about patient health (*M* = 4.7), gender differences (*M* = 4.7), and lack of social acceptability (*M* = 4.6). Community pharmacists in KSA possessed positive attitudes, professional education, and willingness to provide counselling to patients on sex education and reproductive health. Apart from the existing barriers that require augmented community pharmacists’ soft skills, clear policies and authorization for offering this type of service are also needed.

## 1. Introduction

The promotion of overall health, particularly within the context of medication use, is a core role of community pharmacists [1]. This has traditionally involved responsibilities such as supporting medication adherence, counselling about dosage regimens, and the provision of instructions regarding the side effects of both prescription and over-the-counter (OTC) drugs [2]. While pharmacists have also been involved in health improvement beyond just the use of medications, the focus on this element of their practice has received significant attention recently [3,4]. Specifically, the promotion of sexual and reproductive health services through pharmacies is considered an important responsibility. The recognition of sexual and reproductive health needs has continually increased through modern and traditional societal practices by inhabitants of the Kingdom of Saudi Arabia (KSA) [5,6]. Pharmacists provide medical compounds for hormone replacement, counsel patients on medication designed for reproductive health, and educate patients on the proper use of contraception and avoidance of sexually transmitted infections (STIs) [7,8,9]. Recently, researchers reported an expansion of the pharmacist’s role to include expedited partner therapy, participation in intimate partner violence screening, and dispensing of antibiotics to sexual partners with medical assessment and referrals [3,7].

The acceptance of the idea that sexual health is part and parcel of reproductive health that comprises healthy sexual development, free from disease, disability, and violence, is important for public health advancement [8]. The global population is projected to reach about 8.9 billion by the year 2050. Therefore, sexual and reproductive health as part of public health will continue to impact individuals globally [9].

KSA is rapidly developing as a nation and has a considerable amount of human and economic resources. The country has a high rate of marriage compared with other Arabic-speaking nations [10,11]. It has no minimum legal age for marriage, and many girls are married as soon as they reach menarche. The rate of teenage marriages, which is described as marriage below the age of 16, in Jeddah—a city in the Hejaz region of KSA and the country’s commercial center—reached 27.2% in the year 2000 [12]. The practice is widespread within Arab nations where families have a preference for marrying off their daughters early, with the primary reasons being culture, traditional values, virginity, and family honor [2]. Therefore, with such cultural beliefs and practices in the country, there is a need for more controlled sexual and reproductive health promotion. A royal decree sanctioned a health Act to ensure the provision of comprehensive, equal, accessible, and organized health care services to the whole population [13].

Despite the apparent potential and unique positions that pharmacists have in public health, changes in behavior and perceptions are required to ensure successful service provision [14]. Pharmacists are obligated to accept their role of providing public health services, including those related to sexual and reproductive health, with changed behavior and perceptions towards service provision [15,16,17].

Pharmacist participation in reproductive health care suggests that pharmacists have a major impact on improved access to sexual education and reproductive health services [18]. The legal reclassification of emergency contraceptives (ECs) placed pharmacists at the front line of EC education and delivery, and led to pharmacists practicing in independent community pharmacies providing increased access to ECs [7,10]. Community pharmacies are also well positioned to provide these services as patients do not need to make an appointment before visiting, most pharmacies are open for extended hours, and pharmacists offer confidential services.

A limited number of studies report pharmacists’ involvement in menopause prescription and counselling, abortions, and healthy sexuality promotion. Thongmixay et al. noted that pharmacists play an integral role in the promotion of healthy sexuality and serve as primary consultants in areas of sexual dysfunction, lactation, and menstruation-related disorders [18,19]. El-Mowafi noted that these areas were among the daily practice for many pharmacists in the Middle East [20]. However, evidence from research suggests that pharmacists are underutilized in disseminating information to the public on hormone replacement therapy for menopause management. In addition, while pharmacists are reported to have educated the public on the importance of drug adherence in the management of menopause, their participation remains minimal.

Counselling practices and the quality of sex education offered by community pharmacists are currently under active examination in different regions of the world [21]. However, barriers such as lack of confidence in dispensing and counselling adolescents, concerns over the impact and implication of providing ECs without a prescription, unwillingness to dispense due to religious affiliation, and lack of comfort while discussing sexual health matters have been numerously cited in the literature [22].

### Objectives

Sex education and reproductive health in KSA have been subject to a great deal of research. However, there has been minimal research on community pharmacists’ perceptions and barriers that impact their willingness to offer these public health services [23,24]. This study sought to close this gap by investigating the perceptions of community pharmacists on the provision of sex education and reproductive health services. There are no previous studies in KSA that have directly focused on community pharmacists’ perceptions, barriers, and willingness for offering such services.

## 2. Methods

### 2.1. Study Design and Sampling

This study utilized a cross-sectional survey design [25]. The study was carried out between September 2019 and March 2020 across all regions of KSA. The target population for the study was all pharmacists and pharmacy technicians working in community pharmacies in the various regions of the Kingdom. Alruthia et al. [24] reported more than 24,395 licensed pharmacists and pharmacy technicians employed in KSA. The study utilized convenience sampling, a technique in which each member of the target population is selected in an ad hoc fashion based on individual accessibility coupled with their proximity to the research [26,27]. The sampling procedure was used to invite community pharmacist and technicians from all the major cities of the kingdom. A total of 1323 community pharmacists and technicians were invited to participate in the study. The needed sample size to allow for the generalization of findings at a 95% confidence level was calculated at 378 [28].

### 2.2. Inclusion Criteria

The study considered all community pharmacists and pharmacy technicians between the working ages of 23 to 65 years old that were registered to work at the time of the study, regardless of gender or nationality. In addition to pharmacists, pharmacy technicians were included as part of the target population because they have some experiences that allow them to provide some useful insights towards the understanding of the study topic.

### 2.3. Survey Development

The survey was self-developed to capture the required information to answer the research questions. The survey was divided into four sections. The first section described the demographic characteristics of the target population, which included age, gender, level of education, years of practice, area of residence, and practice setting. The remaining sections explored the independent variables of the study, including the perception of the target population about the provision of sex education and reproductive health, and the proficiency of the target population in providing sex education and reproductive health services.

Perceptions about the provision of sex education and reproductive health were captured using a Likert scale response format. Perceptions about barriers to the provision of sexual and reproductive health services were captured by developing a list of 10 items representing the most cited barriers to sexual and reproductive health service provision. Respondents were asked to rate the intensity of the barrier on a five-point scale (Strongly Disagree (1), Disagree (2), Neutral (3), Agree (4), and Strongly Agree (5)). The five-point Likert scale was used as it is the most recommended by researchers and has been found to reduce the respondents’ frustration levels while improving response rate and quality [25]. The perception of community pharmacists on offering sex education and reproductive health services was measured by the practice of counselling in their daily operations. The research placed a lot of emphasis on counselling on reproductive health, and the feeling towards one of the most popularly utilized reproductive health products: hormonal contraceptives.

The survey instruments were translated from English to Arabic (the mother tongue of most of the participants) and presented to a committee of independent experts for review and verification of the reliability and validity of the survey. Experts were mostly academics, including three professors from the Department of Clinical Pharmacy in the College of Pharmacy at Tiaf University, KSA [25]. In addition, a pilot study was conducted among 52 community pharmacists from the target group to test the quality and simplicity of the research instruments. These 52 pilot participants were volunteers from different cities to ensure diversity. Such tests were also conducted to detect the time needed to complete the survey and assess other difficulties with survey administration and completion. The pilot study revealed that some of the questions were not clear for some of the participants. These questions were subsequently edited to improve readability and understanding.

### 2.4. Data Collection

Once research instruments were developed, they were imported into Google Forms and distributed electronically to the target population. Email contact information was obtained from heads of human resources at the main community chains and from the Saudi FDA website for single pharmacy owners. The survey was sent to a total of 1323 community pharmacists working in different regions across the country. An electronic copy of the survey is provided (https://docs.google.com/forms/d/1GgKvC6BJ6feGDxvB1TTnngR1mTFbSH_elxZbUXULfco/edit?usp=sharing, accessed on 30 September 2020).

### 2.5. Data Analysis

Survey responses were organized and entered into Statistical Package for Social Sciences (SPSS) version 26 for data analysis [29]. Demographic characteristics were computed using descriptive statistics. The perception of community pharmacists was illustrated through histograms and pie charts. The proficiency of community pharmacists to provide sex education and reproductive health services was captured through frequency tables. Additionally, the study used the Cronbach’s alpha coefficient test to measure the internal consistency of the survey.

### 2.6. Ethical Considerations

This study was conducted within the ethical boundaries set by the Saudi Law of Ethics of Research on Living Creatures. Ethical approval to conduct the research was issued from the University Ethics Board before conducting the actual research under reference number 42-0029. The survey instruments were accompanied by a written consent letter from participants to participate in the study.

## 3. Results

### 3.1. Demographics

A total of 1097 community pharmacists responded to the survey (82.9% response rate), which exceeded the desired sample size. Additionally, the reliability of the Likert scale question in the instrument showed an acceptable level of reliability with a Cronbach alpha value of 0.799. The profile of the respondents is shown in Table 1. Most of the respondents were young pharmacists below the age of 35 (*n* = 856, 78%). The industry is male-dominated as 96.7% (*n* = 1060) of the sampled population were males. Most of the community pharmacists sampled in this study had Bachelor’s degrees (*n* = 978, 89.2%). There was a relatively equal distribution of the regions represented, with 38% (*n* = 416) from the western region of KSA, 23.8% (*n* = 261) from the central region, 19.1% (*n* = 210) from the southern region, 9.6% (*n* = 105) from the eastern region, and 9.5% (*n* = 104) from the northern regions. A large majority of pharmacists (*n* = 822, 75%) did not have the Saudi Board certification despite having credible academic qualifications. In terms of their professional levels, 71.2% (*n* = 781) considered themselves pharmacists, 26.3% (*n* = 289) senior pharmacists, 2.4% (*n* = 26) consultant pharmacists, and 0.1% (*n* = 1) technicians. Nearly 76% (*n* = 833) of the sampled population were married. Nearly all respondents (*n* = 1018, 92.8%) were non-Saudi. Chain pharmacies were the most common workplace (*n* = 1092, 99.5%). Most of the community pharmacists were on full-time employment contracts (*n* = 1052, 95.9%). Most respondents were experienced, with the most frequent level of experience being between 6 to 10 years (*n* = 474, 43.2%).

### 3.2. Perceptions on Offering Sex Education and Reproductive Health Services

**Figure 1:**
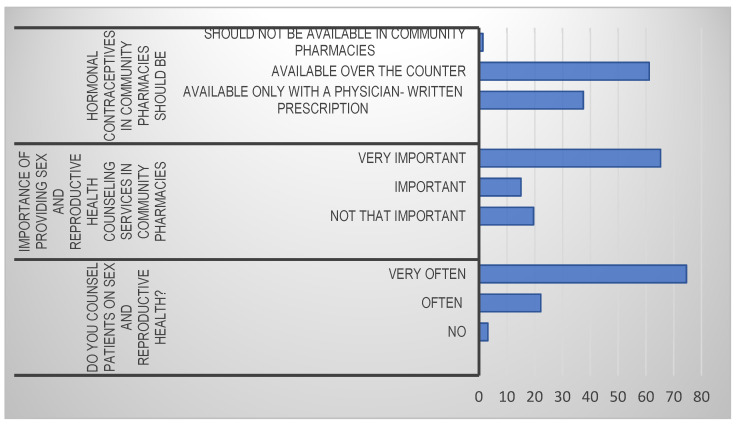
View of community pharmacists’ perception on providing counselling services on sex and reproductive health.

The results in Figure 1 show that the practice of counselling was very popular among community pharmacists. Over 90% of the sampled community pharmacists counselled their patients very often (*n* = 818, 74.6%) or often (*n* = 244, 22.2%), while only 3.2% (*n* = 35) of pharmacists did not counsel patients on sex education and reproductive health. Community pharmacists in KSA viewed counselling of patients on sex and reproductive health as an important element of their work. When asked to rate the importance of providing counselling services on sex and reproductive health, 65.3% (*n* = 716) believed it was very important, 15.1% (*n* = 166) believed it was important, while a minority (*n* = 215, 19.6%) thought it was not that important. Overall, over 80% (*n* = 880) of the sample population placed a high value on counselling patients on sex and reproductive health.

Community pharmacists were also asked to indicate the most common approach to counselling patients. Direct contact or face-to-face consultation were most common (*n* = 563, 51.3%), followed by emails (*n* = 265, 24.2%), leaflets, brochures or posters (*n* = 192, 17.5%), and telephone calls (*n* = 77, 7.0%).

### 3.3. Barriers to the Provision of Sex Education and Reproductive Health Services

Workload was not considered a hindering factor to sex education and reproductive health service provision, with only 15% (*n* = 165) of respondents believing that workload was a hindering factor. Lack of information about patients was a major barrier with 96% (*n* = 1053) of the sample population either agreeing or strongly agreeing. Nearly all community pharmacists (*n* = 1064, 97%) confirmed that fear of responsibility and liability issues was a major barrier to the provision of sex education and reproductive health services. Patient behavior was one of the lower-rated factors hindering the provision of reproductive health services among community pharmacists, with 56% (*n* = 614) of the surveyed population either strongly disagreeing or disagreeing that this was a barrier. Social acceptability was highly rated as a barrier with 85% (*n* = 932) either agreeing or strongly agreeing. Finally, gender difference was identified as a major barrier of reproductive health education with a total of 93% (*n* = 1020) of respondents agreeing or strongly agreeing.

According to the information in Table 2, the results, therefore, show that the factors that most community pharmacists consider as barriers are incomplete patient records, fear of responsibility that may result in liability, a lack of social responsibility, and gender differences. On the other hand, both workload and impatient behavior by patients have little impact as barriers to service provision by community pharmacists.

### 3.4. Proficiency

The survey instruments measured pharmacists’ education and competency levels on sex education and reproductive health, level of preparedness to offer sex education and reproductive health services, level of expertise in emergency contraceptives, and knowledge on the nature of sex education and reproductive health services sought by consumers. When asked to rate their competency level to provide sex education and reproductive health services, nearly half of the respondents reported a very high level of competency (*n* = 507, 46.3%).

Preparedness to participate in and promote sex education and reproductive health as well as the interest to continue getting a basic education in reproductive health were also seen as subsets of proficiency in education. For these subsets, 76% (*n* = 834) were prepared to act as sex education and reproductive health educational promoters, 20% were not sure, while 4% (*n* = 44) were not willing. When it came to willingness to participate in sex education and reproductive health capacity-building programs, 91% (*n* = 998) of the community pharmacists were willing to augment their knowledge on sex education and reproductive health, 6% (*n* = 66) were not sure, while 3% (*n* = 33) were not willing.

When a descriptive analysis of three primary factors was conducted, it was apparent that community pharmacists in the country demonstrated the capacity to provide quality sex education and reproductive health services. For instance, attending sex education and reproductive health educational sessions was a common practice indicating the presence of capacity-building efforts toward improving proficiency in sex education and reproductive health. Most respondents (*n* = 711, 64.8%) said they attended educational sessions on sex education and reproductive health. A large majority of respondents (*n* = 895, 81.6%) reported to have counselled patients on sex and reproductive health. Although the practice of seeking information about a patient’s sexual and reproductive health situations was lowly rated among these factors, an above-average number of respondents (*n* = 638, 58.2%) reported having engaged in this practice.

## 4. Discussion

The importance of sex and reproductive health from a public health point of view has been extensively established in contemporary literature. For instance, the International Covenant on Economic, Social and Cultural Rights (CESCR) considers access to easily available and affordable health services for women as one of the major components of basic rights for women. According to the World Health Organization, over 800 women die every day as a result of simple and treatable sexual and reproductive health complications [24]. Factors shown to reduce sexual and reproductive health-related complications include improved access to maternal health services [25], presence of skilled health care professionals [26,27], quality postnatal and neonatal care, availability of family planning, and improved women’s general education and neonatal health [28,29,30].

### 4.1. Perceptions and Barriers

Pharmacists have traditionally played a critical role in providing reproductive health services in KSA. A large proportion of reproductive health pills, condoms, and other reproductive health care services are obtained from community pharmacies. Dispensing of reproductive health care products, either accompanied by informed counselling on their use or not, has not been measured effectively in KSA. Findings from this study established that community pharmacists viewed the practice of counselling on sex education and reproductive health care services as very important (Figure 1). The results of this study mirror other studies in different regions of the world that identified positive attitudes towards the practice of counselling on sex and reproductive health care among community pharmacists [19,31].

Contemporary literature cites a wide range of factors that affect the provision of counselling on sexual education and reproductive care. For instance, Tian found that gender inequality and a fragmented health care system were the leading factors preventing successful sexual education and reproductive health care in Nepal; specifically, access to health care and family planning services [32]. Consequently, studies by Ambruoso [33] and Michael found that the cost of health care was equally significant in preventing the provision of extra services such as counselling for sexual education and general reproductive health care [31,33]. Most previous studies were conducted from the perspective of the consumer, and very few studies were identified that examined the question of counselling from the community pharmacist’s perspective [33]. This study established that providing counselling on sex education and reproductive health was the ambition of most community pharmacists in KSA; however, several barriers prevented them from providing these services. Barriers in this study differed from those of prior studies, with fear for responsibility and liability, lack of information about patient health, gender differences, and lack of social acceptability ranked as the top barriers.

Moreover, socio-cultural factors have multiple influences on the provision of sexual education and reproductive health care services. Such factors affect women’s decisions on when and how to receive reproductive health services and influence how health care professionals, in general, deliver their reproductive health care to women. Ambruoso [33] found that a woman’s decision to receive reproductive health care and follow-up treatment depended on the advice of their friends, availability of known personal or family members in a particular health care facility, and concerns over privacy and confidentiality [33]. A study conducted by Al-Zahrani found that the gender of the health care professional was an important consideration for women when seeking reproductive health services [34], where most women tended to prefer female health care providers. There are a limited number of studies that focus on access to reproductive health services in KSA. Compounding this problem is the lack of studies examining the factors that influence pharmacists’ ability to provide reproductive health care services to women. In one of the most popular studies conducted by Al-Zahrani, it was established that women refrained from visiting the postpartum clinic as a result of transportation problems, lack of designated male companion, lack of permission from the husband, and fear of lack of female health care providers in reproductive health services [34]. The findings in this study also found a disproportionate distribution of pharmacists, with the majority being male. The self-assessed proficiency notwithstanding, the disproportionate representation of male pharmacists in the profession is difficult to overcome unless initiatives are set in place to train more female pharmacists.

### 4.2. Proficiency

The provision of quality counselling services irrespective of the barriers and the willingness to offer such services will depend on the proficiency of the health care professional. Proficiency is largely determined by the knowledge and the willingness to continue acquiring more knowledge on a subject of expertise. In this study, proficiency was evaluated by ratings on education and competency levels, preparedness to offer sex education and reproductive health services, level of expertise in emergency contraceptives, and knowledge on the nature of sex education and reproductive health services sought by consumers. The results confirmed that—based on self-assessment—community pharmacists were proficient as a large majority believed they had the proper educational qualification to offer quality counselling services on sex education and reproductive health. Most community pharmacists were confident in their sex education and reproductive health knowledge.

### 4.3. Study Limitations

There were a few limitations to this study, including the use of self-assessed data on competency and knowledge regarding the study topic instead of an actual evaluation of these factors using practical tools and observations. More importantly, details on the pharmacists’ interventions were not collected, and the outcomes of pharmacists’ interventions on patients were not quantified.

Although the study offers insight into the perception, attitudes, and willingness of community pharmacists to offer sexual reproductive consultation services to patients in KSA only, its results may be transferable to the greater region of the Middle East as the cultural practices in the region are largely similar. However, due to the use of convenience sampling, full generalizability is uncertain. Further, results are limited by their scope to chain and independent pharmacies. Government pharmacies make up a substantial proportion of pharmacies in most other countries in the Middle East and as such, more people are likely to visit them as opposed to private establishments.

## 5. Conclusions

This study established that there are significant gaps in community pharmacists’ perceptions and barriers that influence their willingness to offer services about sexual and reproductive health. The most significant reasons include workload, lack of supportive policies, privacy concerns, social acceptability, and lack of important information about a patient’s health. It is also important that relevant stakeholders (such as the Pharmacists Board) create ways to enable pharmacists to provide quality counselling services regarding sexual and reproductive health irrespective of the existing barriers for the benefit of the society at large. Despite the existing barriers, community pharmacists showed that they have taken an interest in sex and reproductive health education, offer counselling services to their patients, and seek information from their patients regarding their sexual and reproductive health. This is encouraging as it shows positive steps towards a better understanding and attitude towards offering sexual and reproductive health services.

## Figures and Tables

**Table 1:** Demographic characteristics of survey respondents.

Descriptive Characteristics of the Respondents		
Particulars	Frequency	Percent
Age		
22–34 years	856	78.0
35–44 years	218	19.9
45–54 years	23	2.1
Gender		
Female	36	3.3
Male	1061	96.7
Work location		
In a city	1006	91.7
In a village	91	8.3
Qualification		
Bachelor	978	89.2
Diploma	6	0.5
Fellowship or residency program	1	0.1
Masters	9	0.8
Pharm.D.	103	9.4
Working region		
Central region	261	23.8
Eastern region	105	9.6
Northern region	104	9.5
Southern region	210	19.1
Western region	417	38.0
Board certification		
No	823	75.0
Yes	274	25.0
Present professional level		
Consultant pharmacist	26	2.4
Pharmacist	781	71.2
Senior pharmacist	289	26.3
Technician	1	0.1
Marital status		
Married	831	75.8
Single	261	23.8
Nationality		
Non-Saudi	1018	92.8
Saudi	79	7.2
Work place		
Chain pharmacy	1092	99.5
Independent pharmacy	5	0.5
Employment contract status		
Full-time	1052	95.9
Part-time	45	4.1
Years of experience		
<2 years	63	5.7
2–5 years	292	26.6
6–10 years	474	43.2
>10 years	268	24.4

**Table 2:** Barriers to offering sexual and reproductive health services among community pharmacists.

*N*	Total Number of Responses (1097)						
	Item	Percentages					Mean
		Strongly Disagree	Disagree	Neutral	Agree	Strongly Agree	
1	Workload	7	70	3	15	5	2.3
2	Lack of supportive policy	2	6	54	36	2	3.5
3	Lack of privacy during service provision	8	12	1	25	54	3.8
4	Lack of information about patient health	1	1	2	8	88	4.7
5	Fear for responsibility and liability issues	0	1	2	5	92	4.8
6	Impatient behavior of the patient	56	28	0	14	2	1.7
7	Lack of incentives and financial rewards	2	4	34	45	15	3.2
8	Pharmacists not knowledgeable enough	5	12	18	36	27	3.1
9	Lack of social acceptability	2	5	8	10	75	4.6
10	Gender difference	1	3	3	10	83	4.7

## Data Availability

The data that support the findings of this study are available from the corresponding author, upon reasonable request.
